# Positive association between the plasma levels of 5-hydroxyindoleacetic acid and the severity of depression in patients with chronic obstructive pulmonary disease

**DOI:** 10.1186/1471-244X-13-159

**Published:** 2013-05-31

**Authors:** Tomomi Sekiduka-Kumano, Tomotaka Kawayama, Kosuke Ito, Yoshihisa Shoji, Kazuko Matsunaga, Masaki Okamoto, Nobutaka Edakuni, Haruki Imaoka, Naohisa Uchimura, Tomoaki Hoshino

**Affiliations:** 1 Division of Respirology, Neurology, and Rheumatology, Department of Medicine 1, Kurume University School of Medicine, 67 Asahi-machi, Kurume 830-0011, Japan; 2 Department of Neuropsychiatry, Kurume University School of Medicine, 67 Asahi-machi, Kurume 830-0011, Japan

**Keywords:** COPD, Monoamine, Depression

## Abstract

**Background:**

The role of plasma monoamines in patients with chronic obstructive pulmonary disease (COPD) with depression is unclear. To investigate monoamines in 20 depressed patients with COPD, the plasma concentrations of serotonin, 5-hydroxyindoleacetic acid (5-HIAA), homovanillic acid, and 3-methoxy-4-hydroxyphenylglycol (MHPG) were measured and compared with those in 50 non-depressed COPD patients, and also with 23 age- and gender-matched non-smokers and 13 smokers as non-depressed healthy controls.

**Methods:**

Diagnosis of depression was assessed using the Centre for Epidemiologic Studies Depression Scale. Plasma concentrations of monoamines were measured by high-performance liquid chromatography.

**Results:**

None of the depressed COPD patients had suicidal ideation. The plasma 5-HIAA level [median, (25% and 75% quartiles)] in depressed COPD patients [6.8 ng/mL, (4.9 and 13.1)] was significantly higher than in non-depressed COPD patients [5.4, (4.2 and 7.5)] (p=0.022) and non-smokers [5.1 (3.8 and 7.2)] (p=0.041), but not smokers [4.7, (4.0 and 6.7)] (p>0.05). The plasma 5-HIAA level (r=0.24, p=0.049) was significantly associated with the severity of depression in patients with COPD. The plasma MHPG level was significantly higher in depressed COPD patients (p=0.043) than in smokers, but was not higher than that in non-depressed COPD patients or non-smokers, although the level of MHPG was not associated with the severity of depression.

**Conclusion:**

The plasma 5-HIAA level is increased in depressed COPD patients. Plasma monoamines may be a good biomarker for detection of depression in patients with COPD.

## Background

Chronic obstructive pulmonary disease (COPD) is characterized by a chronic airflow limitation, and is recognized as a major health problem responsible for chronic morbidity and mortality worldwide [1]. Symptomatic COPD patients who have suffered previous repeated exacerbations have poor disease control and prognosis [2]. Improvement of symptoms and prevention of exacerbations may contribute to an improvement of health-related quality of life (HRQOL) and lower mortality for patients with COPD.

COPD patients often have psychological disorders, including depression, and such patients tend to have more frequent exacerbations and a poor prognosis [3-7]. Recently, we demonstrated that depressed COPD patients had a lower HRQOL and more frequent exacerbations and hospitalizations those non-depressed COPD patients [3]. The severity of depression in COPD is closely associated with suicidal ideation [8,9].

It is well known that depressive symptoms are associated with dysfunction of brain monoaminergic neurons, and that the levels of serotonin (5-hydroxytryptamine [5-HT]) released in the brain are linked to a decrease in responsiveness to anti-depressants [10,11]. It is also well known that the functions of monoamine and monoamine oxidase are associated with smoking-related diseases [12,13]. However, the relationship between levels of plasma monoamines and their metabolites in patients with COPD-associated depression is still unclear.

In the present study, we analyzed serotonin metabolites to investigate possible biomarkers of depressed COPD patients. Plasma homovanillic acid (HVA), 3-methoxy-4-hydroxyphenylglycol (MHPG), 5-HT, and 5-hydroxyindoleacetic acid (5-HIAA) were measured in COPD patients who were depressed (depressed COPD) and COPD patients who were not depressed (non-depressed COPD), and also in age- and gender-matched non-depressed nonsmokers and smokers as controls.

## Methods

### Participants

The subjects of this study were outpatients or healthy volunteers. We randomly enrolled 70 patients with COPD, and recruited 36 age- and gender-matched healthy controls between September 1^st^ 2009 and August 31^st^ 2011 at the Chest Disease Center of Kurume University Hospital, Japan (Table 1). All of the patients analyzed had had stable COPD for at least 4 weeks prior to blood tests. None had received oral or injective corticosteroids or antibiotics for 4 weeks prior to the blood tests. Individuals with asthma, bronchiectasis, interstitial pneumonia, tuberculosis, pneumoconiosis, ischemic heart disease, chronic heart disease, renal or liver failure, active malignancies of any organs, sleep apnea syndrome, and a presence and history of psychological diseases such as major depression, bipolar disorder, or schizophrenia were excluded. Also excluded were patients who had been taking anti-depressants, and patients who had a history of lung volume reduction surgery, lung transplantation, or pneumonectomy. Patients with central nervous system disorders and cerebrovascular diseases were excluded on the basis of brain computed tomography (CT) or magnetic resonance imaging (MRI) examinations. Patients with COPD who were undertaking respiratory rehabilitation, or receiving long-term oxygen therapy and non-invasive positive pressure ventilation were excluded, because these treatments are thought to affect psychological status. We carefully excluded any subjects with renal function disorders (serum creatinine levels >1.2 mg/dL).

**Table 1:** Profiles of the four participant groups

**Parameter**	**Control subjects**		**COPD patients**	
	**Nonsmokers**	**Smokers**	**Non-depressed**	**Depressed**
Number of subjects	23	13	50	20
Age (yr)	66.7 ± 9.0	66.5 ± 11.6	68.5 ± 7.2	68.2 ± 7.8
Gender^a^ (no. of males; %)	15 (65.2)	12 (92.3)	47 (94.0)	14 (70.0)
Body mass index (kg/m^2^)	22.1 ± 2.4	23.0 ± 2.8	21.9 ± 3.1	19.8 ± 3.8^†^
Smoking status^a^				
Non / Ex / Cu (no.)	23 / 0 / 0	0 / 4 / 9	0 / 33 / 17	0 / 14 / 6
Smoke index (pack-yrs)	0	40.0 ± 18.2^***^	59.3 ± 30.3^***^	52.2 ± 27.0^***^
Comorbidities^a^				
Hypertension (no; %)	7 (30.4)	4 (30.8)	13 (26.0)	4 (20.0)
Diabetes (no; %)	5 (21.7)	1 (7.7)	11 (22.0)	6 (30.0)
Duration of COPD (yr)	N/A	N/A	5.3 ± 3.9	5.5 ± 4.4
GOLD stage^a^				
I / II / III / IV (no.)	N/A	N/A	7 / 22 / 17 / 4	2 / 4 / 8 / 6
Lung function parameters				
Before bronchodilator				
FVC (L)	3.5 ± 0.9	3.6 ± 0.7	3.4 ± 0.8	2.7 ± 0.9^*†‡^
%FVC	108.5 ± 17.0	105.6 ± 19.3	98.8 ± 18.3	86.4 ± 22.2^**†^
FEV_1_ (L)	2.6 ± 0.6	2.6 ± 0.5	1.5 ± 0.6^***†††^	1.1 ± 0.7^***†††^
%FEV_1_	100.8 ± 14.3	94.9 ± 20.1	54.9 ± 20.8^***†††^	44.8 ± 24.9^***†††^
FEV_1_/FVC (%)	77.2 ± 6.9	73.8 ± 5.2	44.4 ± 12.7^***†††^	41.0 ± 16.2^***†††^
After bronchodilator				
FVC (L)	3.4 ± 0.9	3.5 ± 0.7	3.4 ± 0.8	2.6 ± 1.0^*†‡^
%FVC	108.0 ± 17.4	104.3 ± 18.0	99.3 ± 18.5	84.9 ± 23.5^**†‡^
FEV_1_ (L)	2.7 ± 0.6	2.7 ± 0.4	1.6 ± 0.6^***†††^	1.2 ± 0.7^***†††^
%FEV_1_	103.0 ± 15.5	96.5 ± 19.9	56.4 ± 20.8^***†††^	45.3 ± 25.0^***†††^
FEV_1_ / FVC (%)	79.2 ± 6.5	76.0 ± 5.9	45.6 ± 13.4^***†††^	42.3 ± 15.9^***†††^
Reversibility of FEV_1_ (%)	2.2 ± 4.5	1.8 ± 3.8	3.3 ± 5.3	1.0 ± 5.2
Arterial blood gases				
PaO_2_ (Torr)	90.3 ± 7.5	92.9 ± 5.9	76.6 ± 9.5^***†††^	71.9 ± 13.9^***†††^
PaCO_2_ (Torr)	41.4 ± 3.2	41.7 ± 3.5	40.0 ± 4.0	44.6 ± 7.1^‡^
mMRC dyspnea scale	0.0 ± 0.0	0.2 ± 0.6	1.0 ± 1.1^***†††^	2.1 ± 1.6^***†††§^
SGRQ				
Total score (units)	8.4 ± 8.3	15.7 ± 12.0	32.6 ± 15.9^***††^	57.8 ± 20.8^***†††¶^
Symptom score (units)	19.6 ± 13.4	30.9 ± 18.9	40.8 ± 21.3^**††^	66.2 ± 16.7^***†††¶^
Activity score (units)	6.0 ± 7.1	20.9 ± 17.2	42.9 ± 24.1^***†††^	68.2 ± 29.7^***†††§^
Impact score (units)	6.2 ± 10.0	8.0 ± 9.9	21.3 ± 13.9^**††^	52.0 ± 23.2^***†††¶^
CES-D scale	1.7 ± 2.9	2.5 ± 3.1	8.5 ± 5.2^***†††^	24.5 ± 6.0^***†††¶^
Treatments for COPD^b^				
LAMA (no; %)	0	0	33 (66.0)	16 (80.0)
LABA (no; %)	0	0	21 (42.0)	10 (50.0)
ICS (no; %)	0	0	15 (30.0)	11 (55.5)
SRT (no; %)	0	0	7 (14.0)	4 (20.0)

All data were expressed as mean ± SD and compared by one-way ANOVA and Tukey-Kramer test for multiple comparisons among the four groups.

^a^ Data were compared among groups by chi-squared test for trend.

^b^ Data were compared between depressed and non-depressed COPD patients by Fisher’s exact test. Numbers of non-depressive and depressive COPD patients who used salmeterol and fluticasone devices in combination were 13 and 10, respectively. Some patients were taking multiple medications.

* p<0.05, ** p<0.01, and *** p<0.001 vs. nonsmokers.

† p<0.05, †† p<0.01, and ††† p<0.001 vs. smokers.

‡ p<0.05, § p<0.01, and ¶ p<0.001 vs. non-depressed COPD patients.

Non, non-smokers; Ex, ex-smokers; Cu, current smokers; GOLD, Global Initiative for Chronic Obstructive Lung Disease; FVC, forced expiratory capacity; FEV_1_, forced expiratory volume in 1 second; PaO_2_, partial pressure of arterial oxygen; PaCO_2_, partial pressure of arterial carbon dioxide; mMRC, modified Medical Research Council; SGRQ, St George’s respiratory questionnaire; CES-D, Center for Epidemiological Studies depression scale; LAMA, long-acting muscarinic receptor antagonist; LABA, long-acting β_2_ agonist; ICS, inhaled corticosteroid; SRT, slow-release theophylline; N/A, not available.

As reported previously [14,15], the sample sizes for the patients with COPD and healthy controls were >70 and >35, respectively, in plasma levels of monoamines, when the sample ratio was 1:2 (power = 80%; alpha error = 5%; and beta error = 80%).

### Study protocol

After the patients had provided written informed consent, information on age, gender, smoking status (current-, ex- or non-smoker), cumulative smoking history (pack-yrs), body mass index (BMI; weight/height^2^), comorbidities, and history of pharmacological treatments was obtained. Each subject underwent blood tests, spirometry, electrocardiography, chest radiography, chest high-resolution CT (HRCT), and brain CT or MRI. Spirometry and bronchodilator response tests were performed using an electronic spirometer (Chestgraph Jr HI-101, CHEST Ltd., Tokyo, Japan) in accordance with the American Thoracic Society (ATS) recommendations [16]. A metered-dose salbutamol (400 mcg/subject, GSK, Japan) inhaler was used as a bronchodilator, and bronchodilator response tests were performed before and 30 min after salbutamol inhalation. Predicted values of spirometry parameters were calculated according to the prediction equations of the Japanese Respiratory Society, as we have reported previously [17]. HRQOL was assessed using the validated Japanese St. George’s Respiratory Questionnaire (SGRQ) [18,19]. The SGRQ contains three subscales (symptoms, activity, and impact), and the total score varies from 0 to 100 with a higher score indicating a worse health status [19]. Dyspnea was evaluated using the 5-grade (0 to 4) modified Medical Research Council (mMRC) dyspnea scale [20]. Arterial blood gas analysis was performed with each subject supine breathing room air. After assessing the SGRQ, the mMRC dyspnea scale, and the Centre for Epidemiologic Studies depression (CES-D) scale (Purchased from Saccess Bell Co., Ltd, Japan) for depression, all blood samples were taken between 9:00 and 10:00 AM following 10 minutes with the subjects supine. Samples were kept at -80°C until analysis.

The study protocols (Approval No. 08091, May 29^th^, 2009) were approved by the research ethics board of Kurume University and written informed consent was obtained from the internal review board and all participants.

### Diagnosis and severity of COPD

Diagnosis and staging of COPD were in accordance with the Global Initiative for Chronic Obstructive Lung Disease (GOLD) guidelines [2], and included a post-bronchodilator forced expiratory volume in 1 second/forced vital capacity (FEV_1_/FVC) ratio of <70%, and <200 mL and <12% reversibility of FEV_1_ before and after bronchodilator administration. Patients with COPD who had a smoking history of 10 pack-yrs and over and also had emphysematous changes in the lungs were selected to carefully remove asthmatics. The emphysematous changes were visually recognized as low-attenuation areas by chest HRCT [21].

### Diagnosis of depression

Diagnosis of depression was assessed using the validated Japanese CES-D scale. The cut-off point for depression was CES-D >16 [22,23] and a CES-D score was assessed for each subject. The results of the CES-D score were not opened until this study was completed. Therefore, physicians did not know the psychological conditions of each subject. In this study, patients with a CES-D score of > 16 were a subgroup of “depressed” COPD patients. For the purpose of this study, “depression” means possible “depression” as determined by the CES-D score.

### Measurement of plasma monoamine levels

Plasma levels of 5-HT, 5-HIAA, HVA, and MHPG were measured by high-performance liquid chromatography (SRL Inc., Tokyo, Japan), as previously reported [15,24-26]. The lowest detection limits for 5-HT, 5-HIAA, HVA, and MHPG were 0.01 μg/mL, 1.8 ng/mL, 4.4 ng/mL, and 3.2 ng/mL, respectively, and for statistical analysis one half of the lowest value was assigned if the value was below the detection limit.

### Statistical analyses

Data analyses were performed using JMP version 7 (SAS Institute, Inc., Cary, NC). Data for the subjects were expressed as the mean ± standard deviation (SD), and data for plasma monoamine levels were expressed as the median, and 25% and 75% quartiles. Statistical analyses were performed using parametric Student’s *t* test for comparison between two groups, or one-way analysis of variance (ANOVA) and Tukey-Kramer test for multiple comparisons among four groups. Correlations were analyzed by parametric or non-parametric Spearman’s tests. Differences between groups were evaluated using the chi-squared test for trend and Fisher’s exact test. The level of significance was set at P <0.05.

## Results

### Subject characteristics

A total of 106 subjects participated in the study and the characteristics of the four groups, namely the 23 non-smokers and 13 smokers (as age- and gender-matched non-depressed healthy controls), and 50 non-depressed and 20 depressed COPD patients, are compared in Table 1. The depressed subjects without COPD were not enrolled in the study. None of participants, including the depressed COPD patients, had any history of attempted suicide.

The control subjects were 23 non-, 4 Ex-, and 9 current smokers, whereas the number of non-, Ex-, and current smokers were zero, 57, and 23 in patients with COPD (p<0.001). There was no significant difference in the smoke index among smokers, and non-depressed, and depressed COPD patients. There were no significant difference in the populations of subjects with hypertension and diabetes among nonsmokers, smokers, and, non-depressed, and depressed COPD patients (p>0.05). All the subjects with hypertension had been taking anti-hypertensive medications whereas the numbers of nonsmokers, smokers, non-depressed, and depressed COPD patients taking anti-diabetes medications were 5, 1, 7 and 4, respectively.

In COPD, there was no significantly difference in duration of COPD between non-depressed and depressed COPD patients (p>0.05). The depressed COPD patients trended to have more progressive GOLD stages than the non-depressed patients but there was no significant difference in the populations of GOLD stage I (14% vs 10%), II (44% vs 20%), III (34% vs 40%), and IV (8% vs 30%) between two groups (p>0.05 by Chi-square test for trend).

Lung function tests showed that he depressed COPD patients had significantly lower FVC and %FVC than the non-depressed patients both before and after bronchodilator use, although both depressed and non-depressed COPD patients had significantly lower FEV_1_, %FEV_1_ and FEV_1_/FVC both before and after bronchodilator use than non-smokers and smokers, respectively (all p<0.001). However, there was no difference in the reversibility of FEV_1_ after bronchodilator use among the four groups.

Arterial blood gas analysis showed that the depressed COPD patients (p<0.05) had significantly more severe hypercapnia than the non-depressed patients, whereas both depressed (both, p<0.001) and non-depressed COPD patients (both, p<0.001) had significantly more severe hypoxia than the non-smokers and smokers, respectively.

Depressed COPD patients had significantly higher mMRC dyspnea scales (p<0.05) and lower HRQOL scores (p<0.05) than the non-depressed patients, although both the depressed (both, p<0.001) and non-depressed COPD patients (both, p<0.001) had significantly higher MRC dyspnea scales and lower HRQOL scores than the non-smokers and smokers, respectively.

In managements for COPD, all patients with COPD were receiving vaccinations for seasonal and H1N1 influenza virus and the numbers of depressed and non-depressed COPD patients who had been receiving pneumococcal vaccination within 5 yrs before recruitment were 13 and 8, respectively. There was no significant difference in the ratio of regular use of ICS, LAMA, LABA, and SRT between non-depressed and depressed COPD patients (p=0.061, p=0.387, p=0.601, and p=0.717, respectively. The effects of ICS on psychological and mood status could not be directly determined, as the study was not designed to include a period for wash-out of each controller for COPD.

### Plasma monoamine levels

**Figure 1:**
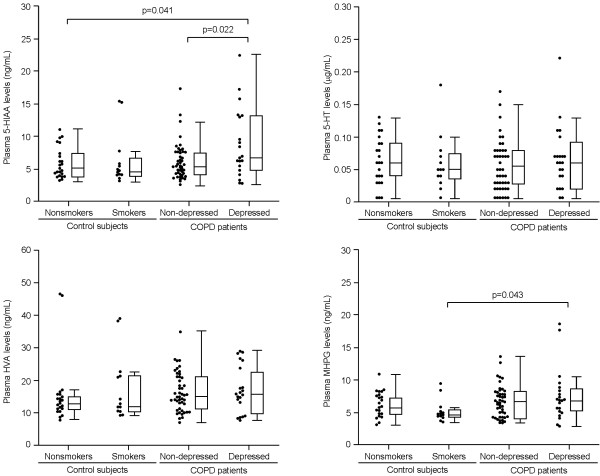
**Plasma monoamines levels.** All data are expressed as plots and boxes with median and 25% and 75% quartiles (error bars 10% to 90%). The numbers of plasma samples taken from non-smokers, smokers, and non-depressed and depressed patients with COPD were 23, 13, 50, and 20, respectively. 5-HIAA, 5-hydroxyindoleacetic acid; 5-HT, serotonin; HVA, homovanillic acid; MHPG, 3-methoxy-4-hydroxyphenylglycol.

Plasma 5-HIAA levels [median, (25% and 75% quartiles)] in the depressed COPD patients [6.8 ng/mL, (4.9 and 13.1)] were significantly higher than in the non-depressed patients [5.4, (4.2 and 7.5)] (p=0.022) and non-smokers [5.1 (3.8 and 7.2)] (p=0.041), respectively, but were not significantly higher than in the smokers [4.7, (4.0 and 6.7)] (Figure 1).

Median (25% and 75% quartiles) plasma 5-HT levels in the non-smokers, smokers, and non-depressed and depressed COPD patients were 0.06 μg/mL (0.04 and 0.09), 0.05 (0.04 and 0.08), 0.06 (0.03 and 0.08), and 0.06 (0.02 and 0.09), respectively. The differences among the four groups were not significant (Figure 1).

Median (25% and 75% quartiles) plasma HVA levels in the non-smokers, smokers, and non-depressed and depressed COPD patients were 12.8 ng/mL (11.0 and 14.8), 11.8 (10.3 and 21.3), 14.8 (11.1 and 20.9), and 15.7 (9.9 and 22.2), respectively. The differences among the four groups were not significant (Figure 1).

Plasma MHPG level [median, (25% and 75% quartiles)] in the depressed COPD patients [6.8 ng/mL, (5.2 and 8.7)] (p=0.043) was significantly higher than in the smokers [4.6, (4.3 and 5.5)]. There was no significant difference in plasma MHPG level between the depressed COPD patients and either non-smokers [5.7 (4.7 and 7.2)] or non-depressed COPD patients [6.7, (4.1 and 8.2)] (p>0.05), respectively (Figure 1).

To investigate seasonal effects in plasma 5-HIAA levels, plasma obtained in four seasons, spring (March-May), summer (June-August), fall (September-November), and winter (December-February), were measured. Number of all subjects and COPD patients in four seasons were 17 and 10, 34 and 27, 25 and 16, and 30 and 17, respectively. There was no significant difference in median plasma 5-HIAA [6.7 ng/mL (5.1 and 7.6) in spring, 5.3 ng/mL (4.3 and 7.4) in summer, 5.9 ng/mL (3.9 and 10.6) in fall, and 5.4 ng/mL (3.7 and 8.2) in winter, respectively, p>0.05].

The plasma levels of 5-HT, 5-HIAA, HVA, and MHPG were not associated with age and there was no significant difference in those plasma levels between male and female.

### Correlation between plasma 5-HIAA level and total CES-D scales in patients with COPD

**Figure 2:**
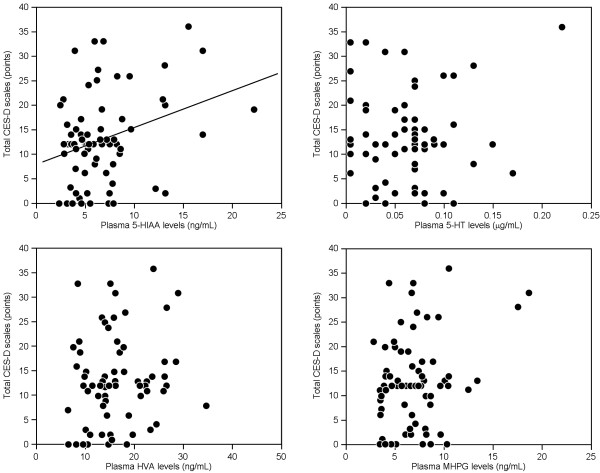
**Correlations between total CES-D scales and plasma monoamines levels in patients with COPD.** Correlations were analyzed using non-parametric Spearman’s test (n=70). CES-D, Centre for Epidemiologic Studies Depression scale; 5-HIAA, 5-hydroxyindoleacetic acid; 5-HT, serotonin; HVA, homovanillic acid; MHPG, 3-methoxy-4-hydroxyphenylglycol.

Plasma level of 5-HIAA (r=0.24, p=0.049), but not that of 5-HT (r=-0.06, p>0.05), HVA (r=0.19, p>0.05), or MHPG (r=0.14, p>0.05), was significantly associated with total CES-D scales in patients with COPD (Figure 2).

### Correlation between plasma 5-HIAA level and BMI, lung function, arterial blood gas parameters, and total SGRQ score in patients with COPD

There was a significant correlation between BMI and plasma MHPG (r=-0.24, p=0.041), but not 5-HIAA (r=-0.21, p>0.05), 5-HT (r=0.07, p>0.05), HVA (r=-0.09, p>0.05), and MHPG (r=-0.24, p=0.041) level in COPD patients.

Plasma 5-HIAA and MHPG level showed significant negative associations with %FVC, %FEV_1_, and partial pressure of arterial oxygen (PaO_2_), and positively associations with partial pressure of arterial carbon dioxide (PaCO_2_) and total SGRQ scores. There was no significant correlation between the plasma 5-HT level and lung function, arterial blood gas parameters, or total SGRQ scores. Plasma HVA level showed a significant negative association with %FEV_1_ and PaO_2_, and a positive association with PaCO_2_ and total SGRQ scores (Table 2).

**Table 2:** Correlations between plasma monoamines levels and lung function, arterial blood gas parameters, and total SGRQ scores in the patients with COPD

	**%FVC, %**	**%FEV**_**1**_**, %**	**PaO**_**2**_**, Torr**	**PaCO**_**2**_**, Torr**	**Total SGRQ, units**
**5-HIAA, ng/mL**	−0.36 (0.002)	−0.40 (<0.001)	−0.38 (<0.001)	0.26 (0.031)	0.33 (0.006)
**5-HT, μg/mL**	−0.09 (NS)	−0.08 (NS)	−0.17 (NS)	0.06 (NS)	0.10 (NS)
**HVA, ng/mL**	−0.36 (0.002)	−0.49 (<0.001)	−0.39 (<0.001)	0.09 (NS)	0.26 (0.029)
**MHPG, ng/mL**	−0.21 (NS)	−0.46 (<0.001)	−0.41 (<0.001)	0.25 (0.036)	0.40 (<0.001)

All correlation coefficients were expressed as r (p value).

Post-bronchodilator data for %FVC and %FEV_1_ were used.

5-HIAA, 5-hydroxyindoleacetic acid; 5-HT, 5-hydroxytryptamine; HVA, homovanillic acid; MHPG, 3-methoxy-4-hydroxyphenylglycol; FVC, forced expiratory capacity; FEV_1_, forced expiratory volume in 1 second; PaO_2_, partial pressure of oxygen; PaCO_2_, partial pressure of carbon dioxide; SGRQ, St. George’s Respiratory Questionnaire.

### Correlation between lung function, total SGRQ scores and total CES-D scales in patients with COPD

The %FEV_1_ showed a significant negative association with total SGRQ scores (r=-0.69, p<0.0001) and total CES-D scales (r=-0.27, p=0.025) in patients with COPD. The PaO_2_ (r=-0.53, p<0.0001) and PaCO_2_ (r=0.44, p=0.0002) showed a significant association with total SGRQ scores. Interestingly, there was no correlation between PaO_2_ (r=-0.21, p>0.05) and PaCO_2_ (r=0.22, p>0.05) and total CES-D scales in COPD patients.

## Discussion

To our knowledge, this is the first study to have measured the plasma levels of monoamines and their metabolites in depressed patients with COPD. Our present results demonstrated that depressed patients with COPD had significantly higher plasma 5-HIAA levels than non-depressed COPD patients and non-smokers. The plasma 5-HIAA levels also showed a significant positive correlation with the severity of depression in the patients with COPD. Our present results support those of a previous study demonstrating that the plasma 5-HIAA levels were significantly increased in patients with depression relative to control subjects, and that the plasma 5-HIAA levels were positively related to the severity of depression [14]. Previous studies have shown that the level of 5-HIAA in cerebrospinal fluid (CSF) was positively associated with the severity of depression in abstinent alcoholics, and that treatment with the antidepressant fluoxetine decreased both the CSF 5-HIAA levels and the mean Hamilton depression rating scale score [27,28]. Other studies have demonstrated that decreased CSF 5-HIAA levels were associated with attempted suicide in patients with depression, and that non-impulsive suicide attempters had higher plasma 5-HIAA levels than impulsive suicide attempters [15,29,30]. In the COPD patients we analyzed, increased plasma 5-HIAA levels were also associated with poor lung function, hypoxia and hypercapnia. Poor lung function is closely correlated with a poor HRQOL, and may result in depression. In COPD patients it has been shown that hypoxia and/or hypercapnia induces oxidative stress and results in an increase of reactive oxygen species (ROS) throughout the whole body including the lungs, brain, and muscles [1-5]. Therefore, it is possible that ROS may have a direct or indirect effect on serotonergic innervation of the lungs, and also affect brain serotonin turnover in the respiratory centres/autonomic centres of the brain. Further analysis will be needed to clarify this issue. None of the participants included in our study had previously attempted suicide. It has been reported that the severity of depression in patients with COPD is closely associated with suicidal ideation [8,9]. Therefore, it would be worthwhile to rank COPD patients in terms of current suicide risk. Taken together, the data suggest that a depressive status and the severity of COPD may be related to increased plasma 5-HIAA level in depressed COPD patients.

The metabolism of 5-HT is controlled exclusively via the action of monoamine oxidase and aldehyde dehydrogenase, resulting in the formation of 5-HIAA. In this study, we did not find any differences in the plasma 5-HT levels among the four groups (non-smokers, smokers, non-depressed COPD and depressed COPD). However, a previous study demonstrated that the plasma 5-HT levels were increased in patients with COPD and was also associated with aging [31]. Further analysis should be needed to verify this issue.

HVA is a dopamine metabolite. Previous studies of patients with depression have demonstrated that the levels of HVA in plasma and CSF decreased along with the levels of 3, 4-dihydroxyphenylacetic acid [14,32,33]. In the present study, plasma HVA levels showed no differences among the four groups we examined. However, the plasma level of HVA showed significant negative associations with %FVC, %FEV_1_, and PaO_2_ levels, and a positive association with the total SGRQ scores in patients with COPD. In COPD patients, most of the HVA in plasma may be derived from precursor dopamine in sympathetic nerves rather than brain dopamine. Our results suggest that poor lung function induced ROS, and perhaps resulted in an increase of HVA derived from precursor dopamine in sympathetic nerves in the COPD patients we studied. Further analysis will be needed to verify this hypothesis.

MHPG is a metabolite of both epinephrine and norepinephrine. Depressed COPD patients had significantly higher plasma MHPG levels (p=0.043) than smokers. Placidi and coworkers [29] suggested that the level of MHPG in CSF might have a positive correlation with aggressive and impulsive suicide, and that selective norepinephrine reuptake inhibitors might increase the risk of suicidal acts. In the present study, however, the plasma level of MHPG was not associated with the severity of depression, but showed significant negative associations with %FEV_1_ and PaO_2_, and positive associations with PaCO_2_ and the total SGRQ score in patients with COPD. Thus, the plasma levels of both HVA and MHPG may be related to the severity of COPD rather than to depression in COPD.

There were some limitations to the present study. First, we measured the levels of monoamines in plasma but not in CSF. Fluctuations in the levels of monoamines and their metabolites can differ between peripheral blood (plasma) and the brain (CSF). Second, the depressed and non-depressed COPD patients were not matched for the severity of COPD, and this parameter may be correlated with plasma monoamine levels. Third, we did not take into account the effects of treatments with antidepressants on the plasma levels of monoamines in depressed COPD patients, although previous studies have reported that antidepressants are of little benefit to patients with COPD [9,34,35]. Further analysis of these issues will be necessary.

## Conclusion

In summary, the present study has shown that plasma 5-HIAA levels are significantly increased in COPD patients with depression, and also associated with the severity of depression in such patients. We also found that the plasma 5-HIAA, MHPG, and HVA levels were negatively associated with lung function, HRQOL and arterial blood gas abnormalities in patients with COPD. Plasma monoamine levels may be applicable as biomarkers for detection of depression in patients with COPD.

## Abbreviations

ANOVA: Analysis of variance; ATS: American thoracic society; BMI: Body mass index; CES-D: Centre for epidemiologic studies depression; COPD: Chronic obstructive pulmonary disease; CSF: Cerebrospinal fluid; CT: Computed tomography; FEV1: Forced expiratory volume in 1 second; FVC: Forced vital capacity; GOLD: Global strategy for diagnosis, management, and prevention of COPD; HRCT: High resolution computed tomography; HRQOL: Health-related quality of life; HVA: Homovanillic acid; ICS: Inhaled corticosteroid; LABA: Long-acting β_2_ agonist; LAMA: Long-acting muscarinic receptor antagonist; MHPG: 3-methoxy-4-hydroxyphenylglycol; MRI: Magnetic resonance imaging; PaCO2: Partial pressure of arterial carbon dioxide; PaO2: Partial pressure of arterial oxygen; SD: Standard deviation; SGRQ: St. George’s respiratory questionnaire; SRT: Slow-release theophylline; mMRC: Modified medical research council; %FEV1: Percent of predicted forced expiratory volume in 1 second; %FVC: Percent of predicted forced vital capacity; 5-HIAA: 5-hydroxyindoleacetic acid; 5-HT: Serotonin (5-hydroxytryptamine).

## Competing interests

This work has no financial competing interests. This work was funded by a grant from the Ministry of Health, Labor and Welfare of Japan (KT), and by a Grant-in-Aid for Scientific Research (C) (no. 21590977: T.H.) from the Ministry of Education, Science, Sports, and Culture of Japan.

## Authors’ contributions

TS-K contributed to protocol design, data collection, analysis, and writing of the manuscript. TK contributed to protocol design and editing of the manuscript. KI contributed to data collection. YS supervised the protocol design. KM contributed to data collection. MO contributed to data collection. NE contributed to data collection. HI contributed to data collection. NU supervised the protocol design and edited the manuscript. TH supervised the protocol design and edited the manuscript. All authors read and approved the final manuscript.

## Pre-publication history

The pre-publication history for this paper can be accessed here:

http://www.biomedcentral.com/1471-244X/13/159/prepub
